# Effect of Moderate Red Meat Intake Compared With Plant-Based Meat Alternative on Psychological Well-Being: A 10-Wk Cluster Randomized Intervention in Healthy Young Adults

**DOI:** 10.1016/j.cdnut.2024.104507

**Published:** 2024-11-16

**Authors:** Tamlin S Conner, Nicola A Gillies, Anna Worthington, Emma N Bermingham, Jillian J Haszard, Scott O Knowles, Daniel R Bernstein, David Cameron-Smith, Andrea J Braakhuis

**Affiliations:** 1 Department of Psychology, University of Otago, Dunedin, New Zealand; 2 Discipline of Nutrition, School of Medical Sciences, Faculty of Medical and Health Sciences, The University of Auckland, Auckland, New Zealand; 3 Fonterra Co-operative, Fonterra Research and Development Center, Palmerston North, New Zealand; 4 Haszard Biostatistics, Auckland, New Zealand; 5 AgResearch Ltd, Smart Foods and Bioproducts Group, Palmerston North, New Zealand; 6 Clinical Nutrition Research Centre (CNRC), Singapore Institute of Food and Biotechnology Innovation (SIFBI), Agency for Science, Technology and Research (A∗STAR), Singapore, Singapore

**Keywords:** dietary intervention, eating behaviors, flexitarian, vegetarian, behavior change, mood, well-being

## Abstract

**Background:**

A healthy diet has been proposed to support good mental health, but the addition of either red meat or meat alternatives is nuanced.

**Objectives:**

We aimed to determine if psychological and physiological well-being is differentially affected by consuming recommended weekly amounts of either lean red meat or plant-based meat alternatives (PBMAs) supplemented with a plant-rich diet.

**Methods:**

The trial was a parallel 2-arm randomized intervention of 10 wk duration. Eighty healthy omnivorous young adults were clustered as 40 cohabitating household pairs. Each pair was randomly assigned to consume 3 weekly servings of either fresh New Zealand beef and lamb or the equivalent PBMA. They maintained an otherwise ovo-lacto vegetarian diet, aided by a weekly meal kit and supported by engaged advice from research dietitians. Psychological measures were well-being (World Health Organization–Five Well-Being Index); depression, anxiety, and stress (depression anxiety stress scales-short form-21); and fatigue (multidimensional fatigue symptom inventory-short form) assessed weekly throughout the trial. Blood biomarkers included neurotransmitter-related compounds, iron status and vitamins B12 and D. Physical activity and sleep were estimated by a fitness wristband. Mixed effect modeling evaluated changes in each outcome over time relative to its baseline and compared the 2 interventions accounting for randomization unit clustering.

**Results:**

Thirty-nine household pairs completed the trial. Participants measured as psychologically healthy at baseline. There were no significant differences between groups in the degree of change from baseline for the psychological outcomes, nor for the majority of the circulatory markers. Differences in changes to vitamin B12 status and 3 neurotransmitter-related compounds (adenosine, agmatine, and tyrosine) from baseline to week 10 were observed between groups. Results were similar in all sensitivity analyses when adjusting for physical activity, sleep, and diet quality covariates.

**Conclusions:**

There was no effect on the psychological measures and limited change to physiological status when comparing a balanced diet containing either red meat or PBMAs in healthy young adults.

## Introduction

The influence that diet has on mental health is complex and multilayered. Up to 20% of the world’s adolescents and young adults have a mental health condition [1], for which diet is a modifiable risk factor [2]. Prospective longitudinal studies and intervention research show that adopting healthy eating such as the plant-rich Mediterranean diet can improve well-being, lower risk of depression, and reduce severity of depressive episodes [[3], [4], [5], [6], [7]]. The quality of the overall dietary pattern is important with unhealthful behaviors including frequent snacking, convenience foods, and inadequate fruit and vegetable consumption, associated with a heightened risk of lower mental well-being [8].

Many consumers are embracing dietary approaches that limit meat intake, such as vegan, vegetarian, flexitarian, and other defined food choice formats [9,10]. The reasons are diverse and include ethical and sustainability concerns, as well as cultural and religious beliefs [[11], [12], [13]]. Whether there is a psychological benefit to abstaining from meat is subject to debate. Some reviews and meta-analyses report significantly higher prevalence of anxiety or depressive symptoms among vegetarians compared with omnivores, albeit with substantial heterogeneity among studies [14,15]. In a meta-analysis including cross-sectional studies on meat abstainers compared with consumers, reported vegans experienced both lower depression and anxiety when compared with meat consumers [16]. Conversely, meta-analysis identifies that risk of depression may increase with intake of red and processed meats [17]. However, both meta-analyses [16,17] include cross-sectional data, and causality cannot be ascribed, which further supports the need for randomized controlled trials, including red meat and meat alternatives. In these and other intervention and survey trials, an effect of meat consumption can be difficult to generalize beyond the test population. This can be participants who are older and following an exercise program [18,19], who are overweight and obese [20], who have prediagnosed symptoms of depression [7], or who have chronic metabolic conditions such as type 2 diabetes [5,21].

The unsettled literature continues with findings of no discernible association between vegetarian diets and psychological outcomes [22,23], and with speculation about reverse causality whereby poor mental health precedes the adoption of a meat-restricted diet [24,25]. Adding to the complexity is an increase in the variety, availability, and appeal of plant-based foods and ingredients that can directly substitute for meat in diets that are otherwise unchanged in composition and quality [26]. Yet, scant research has evaluated this modern phenomenon of plant-based meat alternatives (PBMAs). There are plant-based meat analogs, which are typically highly processed and lack the nutritional complexity of whole, intact foods and are manufactured to look and taste like meat [26,27]. The current investigation used PBMAs including a mixture of meat analogs and soy-based meat alternatives. The red meat was matched to the meat alternatives and provided alongside healthy meals and nutrition support.

Food, mental health, and metabolism are interdependent, and which of these is the cause or consequence is not always clear. While the digestion of food releases compounds and precursors like tryptophan and tyrosine that are involved in neuroregulatory pathways, merely anticipating food can trigger the release of metabolic and neuroactive compounds such as insulin, ghrelin, pancreatic polypeptide, glucagon, and c-peptide, which help regulate appetite and digestion [28,29]. Both mechanisms alter physiology and behavior. The abundance of these bioactive compounds in blood circulation reflects, and can be helpful to distinguish, the various states of mental well-being.

Noninvasive subjective survey instruments are used to assess psychological status. These measure negative aspects such as depression and anxiety and positive aspects such as happiness, meaning, resilience, and finally physical vitality (fatigue, and energy). Historically, research involving dietary association has emphasized the negative aspects of mental health, perhaps missing an opportunity to discover how changes in diet might support optimal psychological functioning [30]. Rarely is a broad range of mental health characteristics considered in nutritional trials.

The objective of our randomized intervention trial was to determine psychological and physiological effects of consuming recommended weekly amounts of either lean red meat or PBMA on top of a plant-rich, ovo-lacto vegetarian diet. The trial intervention lasted 10 wk and involved 80 young adults organized as cohabitating, meal-sharing pairs. Novel approaches to the supervision and support of participants were implemented [31,32].

In this article, we describe the results of the psychological outcomes. Psychological measures of well-being, mental health, and fatigue were collected regularly during clinic visits, remotely between visits and at end-of-trial follow-up. Links to participants’ metabolic response to the dietary intervention were explored by analyzing a selection of blood-borne markers. These included micronutrients and a panel of neurotransmitter-related compounds (NTRCs) that function as precursors and putative modulators of mood.

## Methods

### Trial design and setting

Details about the PRotEin DIet satisfaction (PREDITION) trial design are in our protocol and adherence articles [31,32]. The trial was a 2-group parallel randomized dietary intervention involving consumption of lean red meat compared with a meat substitute. There was a 2-wk pre-allocation period (T-2 to T0) to establish baseline psychological measures and familiarize participant with reporting requirements, without any change to usual diet or lifestyle behaviors. Participants were then notified of their allocation and began receiving their intervention for 10 wk. Participants were contacted again at 22 wk for a remote follow-up assessment (T22). A flow diagram of participation, including screening and exclusions, has previously been published [32]. In brief, 298 recruits were screened for eligibility. Of those, 80 individuals as 40 cohabitating household pairs were enrolled. The pairs were organized into 8 sets of 5 households each. Sets were assigned to the Red meat or PBMA groups using a random allocation sequence (1:1 ratio), yielding 40 individuals as 20 pairs per group. The individuals ranged in age from 18 to 35 y (i.e., GenZ and Millennials). They attended 4 in-person clinics and otherwise engaged remotely and were monitored via frequent email contact and messaging.

The trial was run from the University of Auckland Clinical Research Centre, New Zealand, from May 2021 to August 2022, with 4 cohorts (each comprising 2 sets) sequentially initiated in June, August, February, and March. Some cohorts were disrupted by COVID-19 restrictions, as previously described [32]. A SPIRIT template of scheduling of enrollment, interventions, and assessments are shown in Supplemental Table 1. Studies involving human participants are reviewed and approved by the New Zealand Ministry of Health and Disability Ethics Committee (20/STH/157). Our participants gave written informed consent to take part in this study. Trial design and all primary and secondary outcomes were preregistered at ClinicalTrials.gov as NCT04869163.

### Participants and eligibility criteria

Household pairs were defined as cohabitating individuals, either partners or roommates, who typically prepare and share evening meals. Pairs were recruited together to encourage adherence to the dietary intervention and for practical reasons such as food delivery economies of scale. The recruitment target reflected the sample size necessary to achieve statistical power to test for changes from baseline in the primary outcome between interventions (fatty acid concentration from blood samples; see Braakhuis et al. [31]). Participants needed to be omnivores (consumed ≥2–3 meat-containing meals per week within the last 2 mo including red or white-fleshed meat or seafood) and be willing to consume both red meat and meat analogs during the trial. Participants needed a mobile phone with a camera (used to record diet) and to be comfortable using Facebook and Facebook Messenger (used for communication and behavior support). Exclusion criteria included chronic health conditions, a BMI (in kg/m^2^) ≥ 30, hyperlipidemia, disordered eating patterns, anosmia or ageusia, tobacco use, recreational drug use, medication use (except for contraception and occasional nonsteroidal anti-inflammatory and antihistamine drug use), and pregnancy or planned pregnancy during the trial.

### Dietary intervention and adherence

Participants received a weekly red meat or PBMA allocation (with both participants in a pair consuming the red meat or PBMA intervention), 3 vegetarian meal kits provided by an external supplier, and additional behavior change support in the form of a recipe book, online support, and adherence monitoring [31,32]. The goal was to encourage participants to consume a healthy basal vegetarian diet outside of their allocated red meat or PBMA, while still allowing participants flexibility in their food choices.

Households received regular deliveries of either red meat or PBMA sufficient for 3 dinners for 2 people per week. The quantity of meat was 275–470 g cooked weight per person per week, which could be considered substantial but falls within the latest international recommendations for maximum intake [33]. The meat was fresh New Zealand pasture-raised beef and lamb in the forms of beef mince, beef steak, lamb rack, and lamb leg. The PBMAs were locally available brands of soy- and pea-protein and vegetable oil–based products chosen for their similarity in quantity (3 servings totaling 350–400 g cooked weight), form (mince and patties in a “beef style”), and macronutrient composition (comparable total protein and fat). A range of PBMAs were used throughout the trial, and although fat and protein intake was similar there were differences in micronutrient composition (e.g., some were fortified with vitamins and minerals, others were not).

Both groups could liberally consume eggs and dairy products and other vegetarian protein foods (legumes, nuts, and seeds), but not chicken, pork, fish, or processed meats (e.g., sausage, bacon, salami) to better isolate differences in diets containing red meat or PBMA, and no red meat other than that supplied by the researchers to ensure that participants remained within recommended limits for red meat intake. Participants in both groups were encouraged not to consume PBMAs not provided by the researchers. Participants were provided a recipe book tailored by research dietitians for the PREDITION trial, which guided participants to create quick, easy, and healthy meals containing their PBMA or red meat, and other vegetarian meals to consume across the week.

Households also received a weekly home delivered vegetarian meal kit (Woop Limited, New Zealand), sufficient for 3 meals per week. The meal kits contained pre-prepared meal ingredients (e.g., partially cooked and pre-chopped vegetables, sauces, seasonings) and recipe cards for meals that are quick to prepare, to make healthy eating more accessible with respect to cost, skill level, and time availability. The meals were commercially available, not designed specifically for this trial, and rotated weekly according to the supplier – for example, the first week of meals included mushroom and lentil ragu, hazelnut dukkah halloumi salad, and black bean chipotle mole.

Adherence to the protocol was measured by dietary intake tracking using the Easy Diet Diary app (Xyris Software Pty Ltd), with which participants recorded all meals, snacks, and beverages as a daily record as text (2 d/wk, Sunday and Monday) or photos (5 d/wk, Tuesday to Saturday). Dietary intakes were checked for whether participants consumed the intervention food each week and abstained from other meats or seafood, then this was converted to a weekly score. Individual adherence scores ranged from 65 to 100 and the mean adherence for all participants across the study was 91.5 ± 9.0. There was a high degree of adherence to the protocol in the first 4 wk of intervention, however, the PBMA group dropped at week 7 compared with the Red meat group [[34], [35]].

### Measurements

#### Baseline health, demographic, and anthropometric measures

Health and demographic information was collected from a screening questionnaire including age, sex, ethnicity, highest level of education achieved, frequency and intensity of exercise, and self-rated health from excellent to fair/poor. Participants' height and weight were recorded at the T0 and T10 clinic visits. Height was measured using a stand-alone stadiometer (averaging 2 measurements). Weight was measured using an A&D Scale (HW-PW-200-FG, A&D Medical). BMI was calculated (in kg/m^2^).

#### Psychological measures

The following psychological measures were administered 7 times during the trial: at trial entry (T-2), at end of the pre-intervention period (T0), during the intervention period (T2, T5, T7, and T10), and at the 22-wk follow-up (T22). Measures were completed through an online survey during in-person clinic visits (T-2, T0, T5, and T10) or remotely between clinic visits (T2 and T7) and at follow-up (T22).

Well-being was measured using the World Health Organization–Five Well-Being Index (WHO-5) [36]. This survey has 5 items related to mental and physical quality of life (*I have felt cheerful and in good spirits; I have felt calm and relaxed; I have felt active and vigorous; I woke up feeling fresh and rested; My daily life has been filled with things that interest me).* Participants rated each item for how they felt “over the past week” on a 6-point scale from 0 (at no time) to 5 (all of the time). Note that the original WHO-5 timeframe of “over the last 2 wk” was changed to “over the past week” to standardize the timeframes of all psychological measures in this trial. The 5 items were summed (missing values mean imputed) for a raw score from 0 to 25, which was multiplied by 4 to get a percentage score ranging from 0 (worst possible well-being) to 100 (best possible well-being). Internal scale reliability was very good (mean Cronbach’s *α* = 0.84).

Depression, anxiety, and stress were measured using the depression anxiety stress scales-short form (DASS-21) [37]. This survey has 21 items comprising 37-item subscales measuring depression (e.g., *I felt that I had nothing to look forward to*; *I felt downhearted and blue*), anxiety [e.g., *I felt scared without any good reason*; *I experienced trembling (e.g., in the hands]*), and stress (e.g., *I found it hard to wind down*; *I found myself getting agitated*). Participants rated each item for how they felt “over the past week” on a 4-point scale from 0 (did not apply to me) to 3 (applied to me very much, or most of the time). For each subscale, the 7 items were summed (missing values mean imputed) for a raw score from 0 to 21, which was multiplied by 2 (to be equivalent with the DASS-42) to calculate a final score ranging from 0 to 42 with higher numbers indicating worse depression, anxiety, or stress (mean Cronbach’s *α* = 0.89 for depression, *α* = 0.75 for anxiety, *α* = 0.85 for stress).

Fatigue was measured using the multidimensional fatigue symptom inventory-short form [38]. This survey has 30 items measuring 5 different aspects of fatigue including general, physical, emotional, and mental fatigue as well as vigor (e.g., *I feel fatigued; I feel run down; My body feels heavy all over; I feel tense; I have trouble paying attention; I feel lively,* respectively). Participants rated each item for how they felt “over the past week” on a 5-point scale from 0 (not at all) to 4 (extremely). The items within each subscale were summed (missing values mean imputed) for a subscale score from 0 to 24, then the 5 subscales were added together [(general + physical + emotional + mental) – vigor)] for a total fatigue score ranging from −24 to 96 with higher numbers indicating greater fatigue (mean Cronbach’s *α* = 0.90).

#### Activity and sleep tracking

Participants wore a Huawei Band 4 Pro fitness wristband for the duration of the trial to estimate their level of physical activity and sleep patterns. The device technology includes an accelerometer and heartbeat detection. It was issued at enrollment and participants wore it for 12 wk (from T-2 to T10). Data were transmitted to an app that participants installed on their mobile phones, which yielded estimates of weekly physical activity (in terms of average step count/day) and sleep time (average hours/night) for analysis.

#### Diet quality

Participants completed a self-administered short-form version of the Otago Food Frequency Questionnaire (FFQ) before the intervention (T-2), at T10 (reflecting intake during the intervention), and at the week 22 follow-up (T22). This 57-item semi-quantitative FFQ is validated in New Zealand to assess the nutrient intake of adults [39] and is able to capture the range of foods inherent to flexitarian and ovo-lacto vegetarian diets. Additional free-text entries were included to estimate how many serves of core food groups were consumed each day or week, with standard serving size examples provided. Results of the FFQ have been published [32].

For the current paper the FFQ was consolidated into a healthy diet score. This score measures 4 key dietary behaviors, identified as key behaviors for young adults to improve [33,34]. These are fruit intake per day, vegetables intake per day, soft drinks or energy drinks per week, and types of breads consumed. This score was internally developed; items are derived from 4 of the 15 items of the Healthy Diet Habits Index [40]. Higher scores (to a maximum value of 16) indicate behavior that better aligns with the 2020 New Zealand Food and Nutrition Guidelines for Healthy Adults.

### Blood collection and analysis

Blood samples were collected at T0, T5, and T10 from an antecubital vein of each participant following an overnight fast. Whole blood in an anticoagulant tube was immediately centrifuged and erythrocytes and plasma harvested. Whole blood in a plain tube was coagulated at room temperature for 15 min, then centrifuged to separate serum. All fractions were stored at –80°C until analysis.

Serum biomarkers of iron status (total iron, ferritin, and transferrin saturation) and vitamins B12 and D concentrations were assessed by enzymatic colorimetric assay (Roche Cobas c311).

Plasma NTRC were measured by liquid chromatography-mass spectrometry (LC-MS) of acetonitrile-precipitated plasma samples. These were derivatized with benzoyl chloride and amended with isotopically labeled (^13^C_6_-benzoyl chloride) standards [41]. Chromatographic separation was performed on an ExionLC UHPLC system (AB SCIEX LLC) coupled to an ACQUITY UPLC HSS T3 column (1.8 μm, 1.0 × 100 mm) (Waters Corp), using a 20-min gradient of ammonium formate (10 mM) and formic acid (0.05%) in MilliQ water and acetonitrile. Mass spectrometry data were acquired on a SCIEX 6500+ Triple Quad MS/MS system in positive ion electrospray ionization mode with scheduled multiple reaction monitoring. Data were processed and quantified in SCIEX MultiQuant software (version 3.0.3) and curated by visual inspection of all peaks. From a methodological panel of 50 targeted metabolites, 25 were detected and quantified in the samples. These included polyamines, amino acids, tryptophan- and arginine-derived metabolites, and neurotransmitters including dopamine-derived metabolites.

### Data preparation and statistical analyses

Our protocol for computing baseline scores for the psychological measures was to average the T-2 and T0 surveys. However, for participants in cohorts 3 and 4 we needed to exclude the T0 survey, because it was completed after disclosure of allocation. This was due to COVID-19 restrictions on in-person contact. We then adopted a conservative approach of removing the T0 assessment from cohorts 1 and 2 as well, to ensure that all baseline scores used in the analyses reflected survey data completed before the disclosure of intervention allocation.

All statistical analyses were undertaken in Stata 18.0 (StataCorp). Mean changes [and standard deviation (SD)] from baseline were calculated for each time point for each group. The mean difference in changes between groups along with a 95% CI and *P* value were estimated for each time point using a mixed effects regression model with random effects of household clusters nested within cohort clusters, and adjustment for baseline. Effects were estimated both with and without adjustment for confounders (age, gender, relationship, BMI, and COVID-19 isolation). For comparison between measures using different scales, standardized effect sizes were also calculated using a pooled SD. Analyses were also carried out for the secondary outcomes of step count (a proxy for total physical activity) throughout the intervention, for sleep duration throughout the intervention in those with data at each time point, and for healthy diet score for the T10 time point. These were estimated using the same methods as the primary outcomes, and as there were repeated assessments for step count and sleep duration, an overall estimate across the 10-wk intervention was determined using all measures and an additional nested random effect for participants. Subsequently, further sensitivity analyses on the effect of the intervention on psychological outcomes were undertaken with additional adjustment for step count, sleep duration, and healthy diet score. Missing data were excluded list-wise. Residuals were plotted and examined for homoskedasticity and normality. If the data were right-skewed such that the residuals were affected, then the variables were log-transformed and mean differences back-transformed to represent percent differences. *P* < 0.05 was considered statistically significant. No adjustment for multiple testing was performed and this should be considered when interpreting statistically significant results. Effect sizes and confidence intervals are primarily used for interpretation [42].

For time-course visualizations, mean scores and standard errors (SE) were plotted for well-being, depression, anxiety, stress, and fatigue over time from baseline to follow-up for the 2 interventions.

## Results

From 80 enrollees, 78 individuals as 39 pairs completed the trial (1 pair from the PBMA group was withdrawn at 5 wk due to gastrointestinal discomfort). Demographic characteristics are summarized in Table 1 [43]. Mean age was 25.8 y (SD = 4.3), with slightly more women (55%) than men. A majority (75%) had university education. Most household pairs were in a relationship (61%); the rest were roommates. Most participants lived in areas of moderate deprivation according to the NZDep18 index, and majority of participants worked as professionals or were students (88% total), with the rest working as managers, technicians/trade workers/machinery operators, or sales workers according to the Australian and New Zealand Standard Classification of occupations. Randomly assigned intervention groups were well matched for occupation, deprivation index, relationship, and sex, although more participants in the red meat group had obtained postgraduate university qualifications compared with the PBMA group.

**TABLE 1:** Baseline participant characteristics according to intervention group.

	Total population (*n* = 78)	Red meat group (*n* = 40)	PBMA group (*n* = 38)
Age (y)	25.8 (4.3)	25.9 (3.6)	25.7 (4.9)
Sex			
Female	43 (55%)	25 (63%)	18 (47%)
Male	35 (45%)	15 (38%)	20 (53%)
BMI (kg/m^2^)	23.9 (3.0)	23.5 (2.7)	24.4 (3.2)
Education			
University – postgraduate	23 (30%)	15 (38%)	8 (21%)
University – undergraduate	33 (42%)	17 (43%)	16 (42%)
Secondary school or below	22 (28%)	8 (20%)	14 (37%)
Occupation^1^			
Managers	4 (5%)	0	4 (11%)
Professionals or administrative workers	39 (50%)	23 (57%)	16 (42%)
Technicians, trade workers, and machine operators	3 (4%)	2 (5%)	1 (3%)
Sales workers	2 (3%)	0	2 (5%)
Students	30 (38%)	15 (38%)	15 (39%)
Not employed	0	0	0
Deprivation index^2^			
1–2 (least deprived)	8 (10%)	4 (10%)	4 (11%)
3–4	32 (41%)	16 (40%)	16 (42%)
5–6	18 (23%)	8 (20%)	10 (26%)
7–8	12 (15%)	6 (15%)	6 (16%)
9–10 (most deprived)	8 (10%)	6 (15%)	2 (5%)
Relationship of household unit			
Housemates	30 (39%)	16 (40%)	14 (37%)
Partners	48 (61%)	24 (60%)	24 (63%)
Dietary intake (serves/wk)^3^			
Red meat	2.2 (1.7)	2.1 (1.7)	2.3 (1.7)
Meat alternatives	1.2 (2.5)	1.6 (3.2)	0.8 (1.5)

Data are presented as mean (standard deviation) for continuous variables and number (percentage) for categorical variables.

Abbreviation: PBMA, plant-based meat alternative.

1 Occupation was categorized according to the Australian and New Zealand Standard Classification of occupations (ANZSCO) with an addition of a “student” category.

2 Depreviation was categorized according to the 2018 New Zealand Index of Multiple Deprivation, which measures deprivation at the neighborhood level [43].

3 Derived from the Otago Food Frequency questionnaire.

Baseline values for each psychological variable are presented in Table 2. Participants reported reasonably high baseline well-being, with mean scores of 59.4 and 63.3 for the PBMA and red meat, respectively, where scores >50 indicate greater well-being. Mean scores for baseline depression, anxiety, and stress were in the “normal” range for both groups (reflecting DASS scores < 10 for depression, < 8 for anxiety, and < 15 for stress). Baseline fatigue scores were also low for both groups.

**TABLE 2:** Effects of intervention on well-being, depression, anxiety, stress, and fatigue.

Psychological variable	PBMA		Red meat		Difference in change from baseline^1^, mean (95% CI)	Standardized difference in change from baseline^2^, mean (95% CI)	*P* value
	Change from baseline, mean (SD)						
	n		n				
Well-being							
Baseline	38	59.4 (14.0)	40	63.3 (12.9)			
2 wk	38	0.3 (13.1)	39	2.7 (15.5)	3.6 (−3.2, 10)	0.24 (−0.21, 0.69)	0.296
5 wk	38	3.2 (11.5)	38	−0.8 (15.9)	−3.3 (−13, 6.1)	−0.22 (−0.83, 0.40)	0.492
7 wk	38	2.6 (13.4)	40	−2.3 (15.9)	−3.5 (−11, 4.4)	−0.23 (−0.75, 0.29)	0.381
10 wk	38	3.4 (15.5)	39	2.7 (15.1)	−0.4 (−11, 10)	−0.02 (−0.71, 0.66)	0.943
Mean over 10 wk	38	2.4 (13.4)	40	0.5 (15.6)	−1.4 (−9.2, 6.5)	−0.09 (−0.61, 0.43)	0.736
Follow-up (22 wk)	34	3.1 (12.6)	30	2.5 (16.7)	0.7 (−5.7, 7.2)	0.05 (−0.38, 0.47)	0.823
Depression							
Baseline	38	6.8 (7.5)	40	4.2 (4.2)			
2 wk	38	-0.1 (3.9)	39	−0.1 (3.9)	−0.5 (−2.2, 1.3)	−0.07 (−0.32, 0.19)	0.611
5 wk	38	0.2 (4.8)	38	1.0 (5.2)	0.6 (−2.0, 3.2)	0.09 (−0.29, 0.47)	0.643
7 wk	38	0.3 (5.0)	40	1.0 (5.3)	−0.2 (−2.6, 2.3)	−0.02 (−0.38, 0.33)	0.901
10 wk	38	0.9 (5.0)	39	0.0 (5.5)	−1.4 (−4.2, 1.3)	−0.21 (−0.62, 0.19)	0.306
Mean over 10 wk	38	0.3 (4.7)	40	0.5 (5.0)	−0.2 (−2.2, 1.7)	−0.04 (−0.33, 0.25)	0.811
Follow-up (22 wk)	34	−0.4 (3.5)	30	−0.2 (5.4)	−0.5 (−3.4, 2.4)	−0.08 (−0.51, 0.35)	0.722
Anxiety							
Baseline	38	5.1 (5.6)	40	4.0 (4.5)			
2 wk	38	−1.1 (4.6)	39	−1.7 (3.4)	−1.0 (−2.9, 1.0)	−0.20 (−0.59, 0.20)	0.330
5 wk	38	−0.4 (4.1)	38	−0.6 (3.4)	−0.7 (−2.3, 0.9)	−0.15 (−0.47, 0.18)	0.376
7 wk	38	−0.7 (6.4)	40	−1.0 (4.4)	−0.7 (−3.4, 2.0)	−0.14 (−0.69, 0.42)	0.632
10 wk	38	−1.0 (4.2)	39	−0.9 (4.4)	−0.1 (−2.5, 2.3)	−0.03 (−0.52, 0.47)	0.919
Mean over 10 wk	38	−0.8 (4.9)	40	−1.1 (3.9)	−0.4 (−2.4, 1.5)	−0.09 (−0.50, 0.31)	0.658
Follow-up (22 wk)	34	−0.8 (4.5)	30	−1.7 (3.7)	−1.2 (−3.3, 0.8)	−0.25 (−0.67, 0.17)	0.237
Stress							
Baseline	38	10.9 (6.7)	40	8.9 (7.0)			
2 wk	38	−0.2 (5.2)	39	−1.1 (4.9)	−1.3 (−3.5, 0.8)	−0.18 (−0.47, 0.11)	0.226
5 wk	38	−0.2 (5.4)	38	−0.3 (6.0)	−0.4 (−3.6, 2.7)	−0.06 (−0.49, 0.37)	0.787
7 wk	38	−0.6 (6.9)	40	−0.1 (6.5)	0.2 (−4.0, 4.5)	0.03 (−0.55, 0.61)	0.916
10 wk	38	−1.1 (4.1)	39	−0.5 (5.5)	0.5 (−2.7, 3.8)	0.07 (−0.37, 0.51)	0.749
Mean over 10 wk	38	−0.5 (5.5)	40	−0.5 (5.7)	−0.1 (−3.0, 2.8)	−0.01 (−0.41, 0.38)	0.950
Follow-up (22 wk)	34	−2.1 (4.5)	30	−1.7 (5.1)	0.3 (−2.5, 3.2)	0.05 (−0.34, 0.43)	0.812
Fatigue							
Baseline	38	8.0 (17.8)	40	8.0 (18.7)			
2 wk	38	0.1 (9.8)	39	−3.7 (13.6)	−3.9 (−9.0, 1.3)	−0.21 (−0.50, 0.07)	0.145
5 wk	38	-1.8 (11.8)	38	−1.3 (17.8)	0.9 (−8.7, 10.5)	0.05 (−0.48, 0.57)	0.859
7 wk	38	−0.9 (15.0)	40	−1.1 (15.9)	−0.1 (−8.1, 8.0)	0.00 (−0.44, 0.44)	0.988
10 wk	38	−1.8 (11.0)	39	−1.9 (16.1)	0.5 (−9.5, 10.4)	0.02 (−0.52, 0.57)	0.929
Mean over 10 wk	38	−1.1 (11.9)	40	−2.0 (15.8)	−0.4 (−8.3, 7.5)	−0.02 (−0.45, 0.41)	0.917
Follow-up (22 wk)	34	0.0 (9.6)	30	−5.6 (15.8)	−4.2 (−12.1, 3.8)	−0.23 (−0.66, 0.21)	0.304

Abbreviations: CI, confidence interval; PBMA, plant-based meat alternative; SD, standard deviation.

1 Mean differences (95% CI) in change from baseline for Red meat compared with PBMA were estimated using a mixed effects regression model with random effects for household pair nested within cohort clusters and adjusted for baseline. For mean over 10 wk, an additional random effect for participant was nested within a household.

2 Values standardized into units of SD using a pooled mean and SD (62.7 and 15.2 for well-being; 5.71 and 6.77 for depression; 3.72 and 4.86 for anxiety; 9.22 and 7.34 for stress; and 6.38 and 18.27 for fatigue).

**FIGURE 1:**
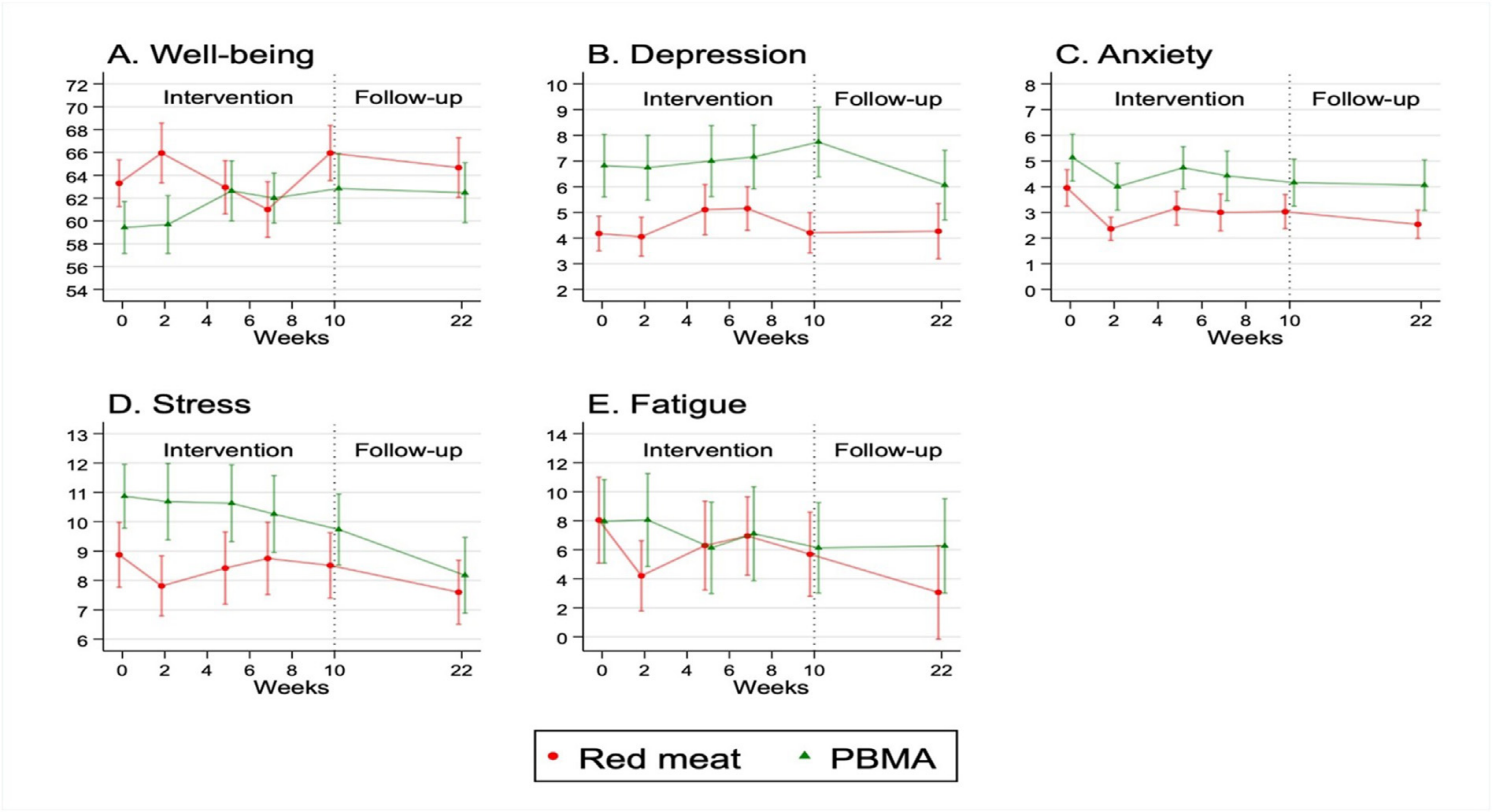
Mean scores of psychological measures of well-being, depression, anxiety, stress, and fatigue by group over the 10-wk intervention (*n* = 78) and at the 22-wk follow-up (*n* = 64). Error bars indicate standard error (SE). Dotted vertical line indicates the end of the intervention period.

Visualizations of changes over time for each psychological variable are shown in Figure 1, with the numeric results presented in Table 2. Mixed effects modeling found no significant differences between the 2 groups in change relative to baseline. Figure 1A suggested a decrease in well-being in the Red meat group and a rise in well-being in the PBMA group at weeks 5 and 7, but these changes from baseline were not statistically different between the dietary groups (Table 2; 5 wk *P* = 0.492, 7 wk *P* = 0.381). Figure 1B revealed that participants in the PBMA group had higher depression scores at baseline and throughout the 10-wk intervention, and a reduction in depression at follow-up (22 wk), but changes from baseline were not statistically different between the dietary groups. Lastly, there was a suggestion of reduced fatigue at 22 wk in the Red meat group compared with the PBMA group (a difference of −4.2 points favoring Red meat; Figure 1E) but this was not statistically significant (Table 2; *P* = 0.304). Notably, all mean differences in change were <0.3 SD with most under 0.1 SD, indicating very small, possibly negligible, differences in mental well-being between the groups. For more detail, Supplemental Figures 1–5 show the time course of well-being, depression, anxiety, stress, and fatigue responses for each participant within their household pair.

Measurements of serum iron status were not significantly different between intervention groups, whereas mixed effect modeling found differences between groups in change from baseline to week 10 (PBMA group −31 ng/L, red meat group + 13 ng/L; difference in change *P* = 0.018) (Table 3). Concentrations in plasma of the NTRC mostly showed no significant differences between groups in their amount of change from baseline, apart from adenosine, agmatine, and tyrosine for which the Red meat group fell and the PBMA group rose. In Table 3 we report values for those 3 plus 6 other detected compounds that are most closely involved in the metabolic pathways of neurotransmitters. Agmatine, glutamate, and 4-aminobutyric acid (GABA) are related to the GABA pathway, tryptophan, kynurenine, and 3-hydroxykynurenine are related to serotonin, and phenylalanine and tyrosine are related to dopamine.

**TABLE 3:** Effects of intervention on blood biomarkers of nutrition and neurotransmitter-related compounds.

Blood biomarker	PBMA, *n* = 37, mean (SD)	Red meat, *n* = 40, mean (SD)	Difference in change from baseline^1^, mean (95% CI)	Standardized difference in change from baseline^2^, mean (95% CI)	*P* value
Serum micronutrients					
Vitamin B12, ng/L					
Baseline	420 (167)	425 (166)			
10 wk change from baseline	−31 (101)	13 (91)	46 (7, 84)	0.29 (0.05, 0.53)	0.018
Vitamin D, μg/L					
Baseline	28.3 (9.8)	26.6 (11.0)			
10 wk change from baseline	−−5.4 (4.5)	−4.4 (4.8)	0.8 (−1.7, 3.3)	0.08 (−0.17, 0.33)	0.521
Iron, μmol/L					
Baseline	17.1 (5.8)	18.9 (7.2)			
10 wk change from baseline	0.6 (6.2)	−0.2 (7.6)	−0.20 (−3.2, 2.8)	−0.03 (−0.47, 0.41)	0.900
Transferrin saturation^3^					
Baseline	0.26 (0.10)	0.29 (0.12)			
10 wk change from baseline	0.01 (0.10)	0.01 (0.12)	0.01 (−0.05, 0.06)	0.01 (−0.07, 0.08)	0.856
Ferritin, mg/L^4^					
Baseline, geometric mean (95% CI)	78.6 (58.5, 105)	69.6 (53.4, 90.8)			
10 wk change from baseline	−0.1 (29.0)	12.2 (34.4)	10.2 (−4.5, 27.2)^4^	—^4^	0.182
Plasma NTRC, nmol/L^5^					
Adenosine					
Baseline	281 (157)	243 (127)			
10 wk change from baseline	43 (211)	−37 (181)	−121 (−186, −55)	−0.85 (−1.31, −0.39)	<0.001
Agmatine^6^					
Baseline, geometric mean (95% CI)	28.8 (26.6, 31.1)	27.0 (25.5, 28.5)			
10 wk change from baseline	1.7 (9.7)	−1.0 (5.6)	−11.4 (−20.3, −1.6)^6^	—^6^	0.024
Glutamate					
Baseline	9608 (3638)	9327 (4826)			
10 wk change from baseline	−204 (4914)	444 (7687)	379 (−1844, 2603)	0.08 (−0.40, 0.56)	0.738
4-Aminobutyric acid (GABA)					
Baseline	62.8 (28.6)	65.7 (16.4)			
10 wk change from baseline	4.0 (29.5)	−2.6 (18.2)	−4.2 (−10.9, 2.4)	−0.22 (−0.56, 0.12)	0.212
Tryptophan					
Baseline	9927 (1815)	9880 (1525)			
10 wk change from baseline	119 (2082)	−99 (1750)	−239 (−1089, 610)	−0.13 (−0.60, 0.34)	0.581
Kynurenine					
Baseline	285 (75)	285 (66)			
10 wk change from baseline	7 (78)	−4 (82)	−11 (−46, 24)	−0.15 (−0.62, 0.32)	0.533
3-Hydroxykynurenine					
Baseline	1101 (388)	1104 (376)			
10 wk change from baseline	19 (556)	−14 (475)	−31 (−210, 148)	−0.08 (−0.54, 0.38)	0.736
Phenylalanine					
Baseline	9468 (1257)	9444 (1440)			
10 wk change from baseline	152 (1549)	−123 (1705)	−290 (−959, 379)	−0.20 (−0.67, 0.26)	0.396
Tyrosine					
Baseline	50,500 (10,390)	51,140 (10,870)			
10 wk change from baseline	3877 (12,190)	−2390 (12,800)	−5903 (−10,940, −857)	−0.53 (−0.99, −0.08)	0.022

Abbreviations: CI, confidence interval; PBMA plant-based meat alternative; SD, standard deviation.

1 Differences in change from baseline for Red meat compared with PBMA were estimated using a mixed effects regression model with random effects for household pair nested within cohort clusters and adjusted for baseline.

2 Values standardized into units of SD using pooled SDs.

3 One participant in the PBMA group had a very high transferrin saturation level at baseline and was removed from this analysis for undue influence.

4 Ferritin values were right-skewed and therefore geometric means and 95% CI are reported for baseline levels, and values log-transformed for analysis so that mean differences are reported as percent differences. Because of this, reporting standardized differences is not relevant. One participant in the Red meat group had a very high ferritin level at 10 wk and was removed from this analysis for undue influence.

5 Agmatine, glutamate, and GABA are related to the GABA metabolic pathway. Tryptophan, kynurenine, and 3-hydroxykynurenine are related to the serotonin pathway. Phenylalanine and tyrosine are related to the dopamine pathway.

6 Agmatine values were right-skewed and therefore geometric means and 95% CI are reported for baseline levels, and values log-transformed for analysis so that mean differences are reported as percent differences. Because of this, reporting standardized differences is not relevant.

Measurements of physical activity (as step count) and sleep (as duration) are presented in Table 4. Daily steps were in the 7000–8000 range, which is at the low end of normative data for habitual activity for this demographic [44]. COVID-19 restrictions may have limited opportunities for activity and exercise for some of the cohorts. There were no significant differences between the 2 groups in steps or sleep relative to their baselines. Healthy diet scores were not different by group or time.

**TABLE 4:** Effects of intervention on physical activity (steps) and sleep duration.

Activity tracking	PBMA		Red meat		Difference in change from baseline^1^, mean (95% CI)	Standardized difference in change from baseline^2^, mean (95% CI)	*P* value
	Change from baseline, mean (SD)						
	N		n				
Steps (count)							
Baseline	35	7721 (3930)	36	7053 (2798)			
2 wk	31	−159 (2826)	36	−806 (2378)	−795 (−201, 429)	−0.20 (−0.52, 0.11)	0.203
5 wk	32	160 (4221)	36	−989 (2929)	−1587 (−3427, 254)	−0.41 (−0.88, 0.07)	0.091
7 wk	28	−154 (3426)	34	−667 (3312)	−713 (−2349, 923)	−0.18 (−0.60, 0.24)	0.393
10 wk	29	−613 (3407)	34	−1213 (2579)	−1011 (−2745, 722)	−0.26 (−0.70, 0.19)	0.253
Sleep (h)							
Baseline	30	7.8 (0.9)	30	7.8 (0.6)			
2 wk	26	0.17 (0.60)	29	0.16 (0.86)	−0.01 (−0.34, 0.32)	−0.01 (−0.41, 0.38)	0.952
5 wk	26	−0.10 (1.22)	27	0.36 (1.05)	0.45 (−0.13, 1.03)	0.53 (−0.16, 1.22)	0.131
7 wk	24	−0.06 (0.71)	27	0.15 (0.98)	0.25 (−0.13, 0.63)	0.30 (−0.15, 0.75)	0.193
10 wk	25	0.16 (0.82)	25	0.20 (0.81)	0.02 (−0.37, 0.40)	0.01 (−0.44, 0.47)	0.931
Healthy diet score	38		40				
Baseline		10.5 (2.6)		10.6 (2.6)			
10 wk		0.9 (2.4)		0.4 (2.4)	−0.3 (−1.7, 1.1)	−0.12 (−0.67, 0.42)	0.657

Abbreviations: CI, confidence interval; PBMA, plant-based meat alternative; SD, standard deviation.

1 Differences in change from baseline for Red meat compared to PBMA were estimated using a mixed effects regression model with random effects for household pair nested within cohort clusters and adjusted for baseline. For the mean over 10 wk, an additional random effect for participant was nested within household.

2 Values standardized into units of SD using a pooled mean and SD.

Sensitivity analyses allowed us to judge how much the estimated effects of the intervention on mental well-being are affected when adjusting for potential confounders (Table 5). There was no appreciable change in results due to covariates or with additional adjustments for physical activity, sleep, or healthy diet score.

**TABLE 5:** Sensitivity analyses for average effects over 10 wk of intervention with covariate adjustment (*n* = 78).

Psychological variable	Unadjusted^1^ (from Table 2)	Adjusted for covariates^2^	Additional adjustment for steps	Additional adjustment for sleep	Additional adjustment for diet score
	Standardized difference in change from baseline^3^, mean (95% CI)				
Well-being					
2 wk	0.24 (−0.21, 0.69)	0.29 (−0.12, 0.69)	0.37 (−0.04, 0.77)	0.23 (−0.20, 0.65)	—
5 wk	−0.22 (−0.83, 0.40)	−0.17 (−0.64, 0.30)	−0.09 (−0.56, 0.38)	−0.12 (−0.58, 0.33)	—
7 wk	−0.23 (−0.75, 0.29)	−0.13 (−0.55, 0.29)	−0.15 (−0.58, 0.28)	−0.15 (−0.56, 0.27)	—
10 wk	−0.02 (−0.71, 0.66)	−0.04 (−0.74, 0.66)	0.15 (−0.61, 0.90)	0.30 (−0.26, 0.86)	0.00 (−0.64, 0.63)
Mean over 10 wk	−0.09 (−0.61, 0.43)	−0.03 (−0.47, 0.41)	0.00 (−0.45, 0.44)	−0.01 (−0.40, 0.39)	0.01 (−0.39, 0.40)
Depression					
2 wk	−0.07 (−0.32, 0.19)	−0.08 (−0.34, 0.17)	−0.08 (−0.32, 0.16)	−0.03 (−0.29, 0.23)	—
5 wk	0.09 (−0.29, 0.47)	0.05 (−0.27, 0.37)	0.04 (−0.31, 0.39)	0.05 (−0.28, 0.38)	—
7 wk	−0.02 (−0.38, 0.33)	−0.06 (−0.41, 0.29)	−0.06 (−0.46, 0.34)	−0.16 (−0.54, 0.23)	—
10 wk	−0.21 (−0.62, 0.19)	−0.29 (−0.63, 0.05)	−0.22 (−0.61, 0.16)	−0.30 (−0.63, 0.04)	−0.31 (−0.65, 0.03)
Mean over 10 wk	−0.04 (−0.33, 0.25)	−0.09 (−0.34, 0.16)	−0.12 (−0.37, 0.13)	−0.13 (−0.37, 0.12)	−0.10 (−0.36, 0.16)
Anxiety					
2 wk	−0.20 (−0.59, 0.20)	−0.19 (−0.57, 0.19)	−0.13 (−0.46, 0.19)	−0.06 (−0.29, 0.18)	—
5 wk	−0.15 (−0.47, 0.18)	−0.12 (−0.47, 0.22)	−0.14 (−0.52, 0.25)	−0.11 (−0.49, 0.27)	—
7 wk	−0.14 (−0.69, 0.42)	−0.11 (−0.67, 0.44)	−0.07 (−0.70, 0.57)	−0.22 (−0.86, 0.42)	—
10 wk	−0.03 (−0.52, 0.47)	−0.08 (−0.55, 0.39)	−0.14 (−0.68, 0.39)	−0.22 (−0.66, 0.22)	−0.10 (−0.56, 0.36)
Mean over 10 wk	−0.09 (−0.50, 0.31)	−0.10 (−0.51, 0.32)	−0.10 (−0.52, 0.33)	−0.11 (−0.49, 0.27)	−0.11 (−0.51, 0.28)
Stress					
2 wk	−0.18 (−0.47, 0.11)	−0.24 (−0.52, 0.05)	−0.24 (−0.52, 0.05)	−0.25 (−0.54, 0.05)	—
5 wk	−0.06 (−0.49, 0.37)	−0.07 (−0.43, 0.30)	−0.12 (−0.51, 0.27)	−0.07 (−0.50, 0.36)	—
7 wk	0.03 (−0.55, 0.61)	0.07 (−0.52, 0.66)	0.02 (−0.63, 0.68)	−0.19 (−0.79, 0.41)	—
10 wk	0.07 (−0.37, 0.51)	0.06 (−0.36, 0.49)	−0.24 (−0.52, 0.05)	−0.25 (−0.54, 0.05)	−0.24 (−0.53, 0.04)
Mean over 10 wk	−0.01 (−0.41, 0.38)	−0.03 (−0.42, 0.35)	−0.06 (−0.45, 0.34)	−0.06 (−0.42, 0.30)	−0.03 (−0.42, 0.35)
Fatigue					
2 wk	−0.21 (−0.50, 0.07)	−0.24 (−0.49, 0.02)	−0.25 (−0.51, 0.01)	−0.15 (−0.44, 0.14)	—
5 wk	0.05 (−0.48, 0.57)	0.04 (−0.43, 0.51)	0.01 (−0.47, 0.50)	0.09 (−0.39, 0.58)	—
7 wk	0.00 (−0.44, 0.44)	0.01 (−0.40, 0.42)	0.04 (−0.43, 0.51)	−0.09 (−0.48, 0.30)	—
10 wk	0.02 (−0.52, 0.57)	0.02 (−0.50, 0.54)	−0.08 (−0.69, 0.52)	−0.14 (−0.61, 0.32)	0.02 (−0.48, 0.52)
Mean over 10 wk	−0.02 (−0.45, 0.41)	−0.04 (−0.44, 0.37)	−0.04 (−0.46, 0.37)	−0.05 (−0.38, 0.28)	−0.05 (−0.42, 0.33)

Abbreviation: CI confidence interval.

1 From Table 2, standardized difference in change from baseline, mean (95% CI).

2 Covariates adjusted for included age, sex, BMI, relationship (partner or roommate), COVID-19 isolation, with cohort clusters included as a random effect.

3 Values were standardized with pooled means and SD (see Table 2). Mean differences (95% CI) in change from baseline for Red meat compared with PBMA were estimated using a mixed effects regression model with random effects for participant nested within household pair nested within intervention group nested within cohort and adjusted for baseline.

## Discussion

This is the first randomized dietary intervention trial to measure the mental health effects of consuming recommended weekly amounts of red meat or PBMA for 10 wk in healthy young adults. We provided meat or PBMA sufficient for 3 dinners per week. For the remaining meals, we supported participants in preparing and consuming a balanced ovo-lacto vegetarian diet. Adherence to this protocol was excellent, with some differences in eating pleasure favoring the red meat intervention [32]. In these participants with above average well-being profiles at baseline, we found no meaningful differences in the change in psychological measures between the Red meat and PBMA groups at any time point throughout the intervention or at the 22-wk follow-up (Table 2). The extent to which physical activity, sleep, and underlying diet quality modified the outcomes was also examined and found to be nonsignificant (Table 4).

The physiological risks and benefits of eating or abstaining from red meat have been often studied, regularly reported, and are usually contradictory. Concomitant effects on psychological health have been less considered. Multiple systematic reviews are contradictory in their support of an altered risk of depression and anxiety in omnivores compared with meat abstainers [[14], [15], [16], [17]], with methodological issues identified regarding dietary recording and recruitment bias, potential modifying factors, small cohort size, and short treatment timeframes. Unaccommodated confounders contribute to the mixed results of the experiments, and they also make it difficult to compare our current results. For example, in a pilot randomized controlled trial of 39 individuals who followed a vegetarian, omnivore, or fish-rich diet for 2 wk [45], short term mood state was affected only by the vegetarian regime (improved). Although the participants were given dietary instructions, that trial did not include targeted behavior modification, evaluate prior diet habits, or monitor sleep and physical activity.

In the PREDITION trial, the objective was to vary just one diet element while moderating the overall health of the remaining diet. However, manipulating any single food or food group can influence nutrition in unexpected ways, with the effect of the test agent entangled in concurrent changes to overall diet quality and composition. For instance, in the National Institutes of Health-American Association of Retired Persons Diet and Health Study (NIH-AARP) of hundreds of thousands of older Americans [46], participants had intakes of unprocessed red meat that ranged as quintiles from 7 to 50 g per 1000 kcal of energy intake. This equates to an absolute intake of 17–125 g/d (by comparison, the PREDITION Red meat group received ∼60 g/d). High meat consumption was associated with greater relative risk of multiple causes of mortality, yet the study also found that meat consumption was correlated with higher total energy and lower fruit and vegetable intakes. As such, biological interactions likely exist.

Similarly, diet quality is an important factor in associations between nutrition and mental health, regardless of whether the diet includes meat [47]. A cross-sectional survey of vegans and vegetarians found that among those without depression, a higher-quality dietary pattern is protective against depressive symptoms [48]. Meta-analyses also found that a “healthier” dietary pattern is inversely correlated with depression [4,49]. Some whole-of-diet interventions that demonstrated significant improvements to mental health have drawn on individuals with diagnosed depression or anxiety [7,50]. In our study, where diet quality was reasonably high and increased over time in both groups [32], including red meat made little difference to mental health outcomes. Our participants were mentally healthy at baseline, with most in the normal ranges for depression (89%) and anxiety (95%). We might expect greater differences in response to diet to emerge with a more at-risk population, including those with pre-clinical or clinical mental health disorders.

Even so, psychological scores did change markedly and nonmonotonically over time, with variance distributed across the individual, household pair, and group (Supplemental Figures 1–5). Individuals within pairs appeared to be more alike than between pairs, suggesting that social cohesion or interaction may have had a role in the measured outcomes. Formal analysis of this interesting sociological and statistical question will need more extensive consideration. At present we have controlled for nonindependent data points by fitting mixed effects models with household pair nested within cohort as a random effect.

Alongside the indices of mental health, we measured blood biomarkers that reflect relevant aspects of nutrition (Table 3). Some micronutrients are responsive to dietary intake and can change as a consequence of meat-inclusive compared with plant-based diets [51,52]. For instance, abstaining from meat products can also limit by 30% the amount of diet-sourced vitamin D [53]. And although cereal products tend to be the largest contributors to iron intake (typically via fortification), animal products are responsible for an additional 20% of intake and importantly, all of the highly bioavailable heme iron.

These micronutrients function as precursors or are putative modulators of mood. Vitamin B12, iron, transferrin saturation, and ferritin are involved in red blood cell physiology and oxygen transport, which relates to feelings of fatigue [54]. Vitamin B12 is also attributed to the regeneration of myelin in peripheral nerves and through one-carbon metabolism to the synthesis of neurotransmitters serotonin and dopamine [55]. Adequate iron status is required to maintain the activity of dopamine-containing brain areas and the density of dopamine receptors, and to support neuronal physiological functions [56]. Vitamin D stimulates transcription of serotonin and is relevant in regulating mood [57]. There were no differences to change in serum iron or vitamin D status between groups, but we did observe differences in changes to serum vitamin B12 concentrations between intervention groups. Serum B12 slightly decreased from baseline in the PBMA group and increased in the red meat group, although the means for both groups at baseline and follow-up were well above deficiency thresholds. The direction of response does align with dietary changes previously reported from this study (see Gillies et al. [32]), which showed that participants’ intake of vitamin B12 significantly decreased during the study by 50% in the PBMA group, although this observed decline in dietary intake according to the FFQ is perhaps exaggerated by not accurately taking all fortified foods consumed into account. Although both intervention groups were able to consume nonmeat sources of vitamin B12 such as eggs and dairy, the decline in vitamin B12 concentrations reflects the known difficulties in meeting requirements without supplementation in plant-based diets. For example, in the large EPIC-Oxford cohort study of European males, 52% of vegans and 7% of vegetarians were found to be deficient in vitamin B12 [58]. Our results do require careful interpretation in light of this fact, as well-formulated plant-based diets should either be adequate from naturally occurring (e.g., eggs, milk), fortified (e.g., plant-based milk alternatives), or supplemental sources of vitamin B12.

Further analysis was performed on a selection of NTRC that may change with diet and alter psychological function or conversely, that mood state influences their circulating abundances (Table 3) [28,29]. The panel of NTRC included compounds that are involved in the metabolic pathways of several neurotransmitters. GABA (panel compounds were agmatine, glutamate, and GABA) plays a role in antidepressant-like action [59]. Serotonin (compounds tryptophan, kynurenine, 3-hydroxykynurenine) has long been implicated in mood and anxiety [60]. The diverse actions of dopamine (tyrosine, phenylalanine) include reward-related processes and the hedonic deficits of depressive disorder [61]. When these were measured, the concentrations of only agmatine and tyrosine (as well as adenosine) were affected by our study interventions (Red meat down, PBMA up). Participants’ dietary intake of these analytes could not be estimated from the FFQ, so the observed responses could be a consequence of either diet or endogenous production. The metabolic synthesis of agmatine, tyrosine, and adenosine is known to be induced by psychological stress and inflammation, and so may trigger changes in neurotransmitter function [[62], [63], [64]]. Overall, the differences in blood biomarker status were minor and unlikely to be of clinical relevance discriminating our PBMA and red meat–supplemented diets. Consistent with this is that functional measures potentially related to altered central nervous system function, including sleep, subjective well-being, and mood, did not change in the study.

There are strengths and limitations in the design and execution of the PREDITION trial. The difficult circumstances of the COVID-19 pandemic had remarkably little impact on participant retention and data collection. This is likely due to implementing robust behavior change framework methodology [33]. Participating in pairs may have also contributed an extra layer of accountability for individuals to engage with the trial. The trial interventions were designed ∼3 meals per week (although some participants stretched their allocation further). This seems like a minor proportion of typical eating patterns yet provided as much red meat as could conscientiously be imposed [35]. Limitations of the trial included the high baseline well-being of participants that limited opportunity for improvement and lack of control for female menstrual cycles that could impact fluctuations in diet and mental states. Similarly, social or personal factors such as romantic breakups, unemployment, and bereavement among others may all significantly impact mental well-being but were not accounted for in participant recruitment or controlled for in data analyses. However, the randomized study design limits the influence of such factors as confounders as these will likely be equal in both intervention groups due to randomization. This was also a “real world” intervention and not a highly controlled context, and the results should be interpreted as such.

Our findings have implications for recommending healthy diets to young adults and for studying the longer-term effects. In particular, the psychosocial aspects of adopting a new eating pattern and sharing the challenges with a partner are important to consider, as they can support success and influence mental well-being. All participants needed to make changes to their diet, albeit toward a healthier eating pattern and more red meat than is usual, however, the shift in diet may have been more difficult for the PBMA group. Another aspect of the trial was that both the red meat and PBMA groups were asked to cut out white meat, fish, and processed meat from their diet for the duration of the trial to minimize modifying factors. Our findings need to be considered within that context. During the 10-wk study, our participants adjusted to an ovo-lacto vegetarian basal diet while adding recommended amounts of red meat or PBMAs. Overall diet quality improved over time and multiple measures of mental health and well-being were equally maintained in both groups.

## Author contributions

The authors’ responsibilities were as follows – TC, AB, NG, SK, EB, and DC-S: designed the research; NG, AW: conducted the research; EB and DB: conducted the biological analysis; JH: conducted the statistical analysis; TC, NG, SK, and AB: drafted the manuscript; TC, AB, and SK: had primary responsibility for the final content and format of the manuscript; and all authors contributed to the article and approved the submitted version.

## Data availability

Data described in the manuscript will be made available upon request pending application and approval.

## Funding

This study and aligned projects were supported by the New Zealand National Science Challenge (High Value Nutrition) and the New Zealand Ministry of Business, Innovation, and Employment including funds from the Meat Industry Association Innovation Limited (a subsidiary of the New Zealand Meat Industry Association) and Beef and Lamb New Zealand Limited.

## Conflict of interest

The study was partially funded by industry trade associations that represent companies supplying almost all of New Zealand meat exports. Those organizations did not have influence on the research design, data collection, or write up. The organizations also had no input into the writing of the publication nor did they have a right of refusal concerning publication (authors can provide contractual evidence to any interested party on request). The data analysis was conducted by an independent biostatistician (JH).
