# Supplementary material for: Effect of Moderate Red Meat Intake Compared With Plant-Based Meat Alternative on Psychological Well-Being: A 10-Wk Cluster Randomized Intervention in Healthy Young Adults
Source: Curr Dev Nutr. 2024 Nov 16;9(1):104507. doi: 10.1016/j.cdnut.2024.104507 (PMC11666937; doi:10.1016/j.cdnut.2024.104507)
Supplement: Multimedia component 1 [file mmc1.docx]

**Effect of moderate red meat intake compared to plant-based meat alternative on psychological well-being: A 10-week cluster randomized intervention in healthy young adults**

**First author: Tamlin S. Conner**

**Supplementary Table 1.** SPIRIT template of scheduling of enrollment, interventions and assessments.

|  |  |  | **Study Period** | | | | | | |
| --- | --- | --- | --- | --- | --- | --- | --- | --- | --- |
|  |  |  | **Lead-in** | | **Post-allocation intervention** | | | | **Follow -up** |
| **Assessments** |  | **Enroll-ment** | **T-2** | **T0** | **T2** | **T5** | **T7** | **T10** | **T22** |
| Eligibility screening |  | X |  |  |  |  |  |  |  |
| Informed consent |  | X |  |  |  |  |  |  |  |
| Demographic information |  | X |  |  |  |  |  |  |  |
| In-person clinics |  | X |  | X |  | X |  | X |  |
| Body mass index |  |  |  | X |  |  |  | X |  |
| Blood measures |  |  |  | X |  | X |  | X |  |
| Diet monitoring |  |  | X | X | X | X | X | X |  |
| Activity/sleep monitoring^1^ |  |  | X | X | XX | XX | XX | XX |  |
| Psychological measures^2^ |  |  | X | X | X | X | X | X | X |

^1^Continuous monitoring from lead-in and throughout the 10-week intervention.

^2^Psychological measures administered at T2, T7, and T22 were done remotely through an online survey.

**Supplementary Figure 1.** Total scores for well-being from the WHO-5 for individuals within their household pair over the course of the 10-week intervention and at the 22-week follow-up. Red indicates Red meat and green indicates PBMA intervention groups.

**Supplementary Figure 2.** Scores from the depression sub scale of DASS-21 for individuals within their household pair over the course of the 10-week intervention and at the 22-week follow-up. Red indicates Red meat and green indicates PBMA intervention groups.

**Supplementary Figure 3.** Scores from the anxiety sub scale of DASS-21 for individuals within their household pair over the course of the 10-week intervention and at the 22-week follow-up. Red indicates Red meat and green indicates PBMA intervention groups.

**Supplementary Figure 4.** Scores from the stress sub scale of DASS-21 for individuals within their household pair over the course of the 10-week intervention and at the 22-week follow-up. Red indicates Red meat and green indicates PBMA intervention groups.

**Supplementary Figure 5.** Total scores for fatigue from the MFSI-SF for individuals within their household pair over the course of the 10-week intervention and at the 22-week follow-up. Red indicates Red meat and green indicates PBMA intervention groups.
